# The Rheumatology Drugs for COVID-19 Management: Which and When?

**DOI:** 10.3390/jcm10040783

**Published:** 2021-02-16

**Authors:** Fabiola Atzeni, Ignazio Francesco Masala, Javier Rodríguez-Carrio, Roberto Ríos-Garcés, Elisabetta Gerratana, Laura La Corte, Manuela Giallanza, Valeria Nucera, Agostino Riva, Gerard Espinosa, Ricard Cervera

**Affiliations:** 1 Rheumatology Unit, Department of Experimental and Internal Medicine, University of Messina, 98100 Messina, Italy; elisabettagerratana@gmail.com (E.G.); lauralacorte7@libero.it (L.L.C.); Manuelagiallanza@outlook.com (M.G.); valerianucera.vn@gmail.com (V.N.); 2 Full Professor, Head of Rheumatology Unit, University of Messina, Via C. Valeria 1, 98100 Messina, Italy; 3 Trauma and Orthopedic Unit, Santissima Trinità Hospital, 09121 Cagliari, Italy; ifm.franc@hotmail.it; 4 Department of Functional Biology, Immunology Area, Faculty of Medicine, University of Oviedo, 33044 Oviedo, Spain; rodriguezcjavier@uniovi.es; 5 Instituto de Investigación Sanitaria del Principado de Asturias (ISPA), 33044 Oviedo, Spain; 6 Department of Autoimmune Diseases, Hospital Clínic, Institut d’Investigacions Biomèdiques August Pi i Sunyer (IDIBAPS), University of Barcelona, 08036 Barcelona, Spain; rober.rg.86@hotmail.com (R.R.-G.); gespino@clinic.cat (G.E.); rcervera@clinic.cat (R.C.); 7 III Division of Infectious Diseases, ASST Fatebenefratelli Sacco, University of Milan, 20127 Milan, Italy; agostino.riva@unimi.it

**Keywords:** pneumonia, COVID-19, SARS-CoV2, anti-IL-6 drugs, HCQ

## Abstract

Introduction: While waiting for the development of specific antiviral therapies and vaccines to effectively neutralize the SARS-CoV2, a relevant therapeutic strategy is to counteract the hyperinflammatory status, characterized by an increase mainly of interleukin (IL)-1β, IL-2, IL-6, IL-7, IL-8, and tumor necrosis factor (TNF)-α, which hallmarks the most severe clinical cases. ‘Repurposing’ immunomodulatory drugs and applying clinical management approved for rheumatic diseases represents a game-changer option. In this article, we will review the drugs that have indication in patients with COVID-19, including corticosteroids, antimalarials, anti-TNF, anti-IL-1, anti-IL-6, baricitinib, intravenous immunoglobulins, and colchicine. The PubMed, Medline, and Cochrane Library databases were searched for English-language papers concerning COVID-19 treatment published between January 2020 and October 2020. Results were summarized as a narrative review due to large heterogeneity among studies. In the absence of specific treatments, the use of immunomodulatory drugs could be advisable in severe COVID-19 patients, but clinical outcomes are still suboptimal. An early detection and treatment of the complications combined with a multidisciplinary approach could allow a better recovery of these patients.

## 1. Introduction

In December 2019, a novel coronavirus was isolated from a group of patients hospitalized with pneumonia, who had connections with a seafood and wildlife market in Wuhan, in the Hubei province of China [1]. The virus was named “severe acute respiratory syndrome coronavirus 2” (SARS-CoV2). The term “Coronavirus disease-19” (COVID-19) was coined by the World Health Organization (WHO) in February 2020 to refer to the related syndrome caused by the virus [2]. The virus is transmitted through droplets and aerosols, which are expelled during the respiratory act or when an infected person coughs, sneezes, or speaks. The most typical clinical picture in SARS-CoV-2 is mainly characterized by upper respiratory infection apparently indistinguishable from any other respiratory infection. However, an exacerbation may occur with a clinical picture of pneumonia (mostly bilateral) characterized by marked dyspnea [3], tachypnea (>30 breaths/min), hypoxemia (SpO2 < 90% in ambient air), and severe lymphopenia with increased acute phase proteins [4], associated with a state of hypercoagulability, an important increase of proinflammatory cytokines interleukin (IL)-2, IL-7, IL-10, G(M)-CSF, IP-10, MCP-1, MIP-1a, TNF, IL-1β, and IL-4 [5]. This uncontrolled pulmonary inflammation is probably one of the main causes of mortality in the severe forms of SARS-CoV-2 infection. Although initially regarded as a ‘cytokine storm’, current evidence does not seem to fully support this classification and this terminology should be used with caution. Cytokine storm syndrome is a relatively ill-define concept, with a number of different triggers and underlying conditions [6,7]. Elevated levels of a broad spectrum of cytokines invited comparisons with other viral infections that lead to dysregulated immune responses, and similarities with other cytokine storm syndromes were proposed [8], such as secondary hemophagocytic lymphohistiocytosis (sHLH) or Macrophage Activation Syndrome (MAS). However, not all severe COVID-19 patients fulfilled sHLH criteria [9], although falling into the umbrella of the phenotype of the hyperferritinemic syndromes. Results from COVID-19 suggest that it may be considered as a unique form of hyperinflammatory response. In this regard, it is important to note that most of the cytokine storm syndromes are relatively rare conditions and derived from non-communicable diseases. Hence, this concept may be difficult to translate to a pandemic-dimensioned infectious disease. Therefore, the idea of the ‘threshold concept’ needs to be considered [10]. Importantly, median IL-6 serum levels are one or two magnitude [11] order lower in COVID-19 compared to fully blown cytokine storm syndromes [8,12], thus supporting that a differential pathogenic scenario is plausible, distinct from the classical cytokine storm framework. However, controversial results have been published elsewhere [6]. Actually, specific criteria have been developed [9] for this condition, hence strengthening the differences observed. Importantly, a mechanism-based rather than clinical context-based taxonomy in cytokine storm is crucial not only for gaining understanding toward pathogenesis but also to better guide the therapy decision-making process [7]. While waiting for the development of specific antiviral therapies, monoclonal neutralizing antibodies and vaccines, which will be able to neutralize the etiological agent, an effective therapeutic strategy could be to counteract the hyperinflammatory status, which is responsible for the most severe clinical cases. In this scenario, the long-lasting experience in rheumatology not only with the use of different immunomodulatory agents but also with disease stratification and complex disease stages’ management could provide valuable therapeutic approaches. In fact, if a timely and effective intervention could be adopted in the moment when the enhanced cytokine release is triggered, the risk for the patient of needing support with invasive mechanical ventilation and developing MAS-like forms would be significantly reduced. However, adaptations from the rheumatology framework are needed in the light of the pathogenic divergences [8]. To date, taking into account published studies, no antiviral or anti-inflammatory treatment has been shown to be clearly effective for SARS-CoV-2 infection and, unfortunately, different studies often show contradictory results. The frenzy to desperately respond to the COVID-19 pandemic prompted a plethora of small-sized, powerless, largely heterogeneous observational trials that have mostly led to inconclusive results on the efficacy of several therapeutic approaches. Too many observational and powerless studies have been conducted and no randomized, double-blind, clinical trials have seen the light. The lack of appropriate control groups, no randomized design, and the different timing of administration of these treatments during infection are the three most important causes that may explain the contradictory findings among studies. Specifically, the critical point in treating the inflammatory phase of COVID-19 is deciding when, how, and to what extent to use anti-inflammatory therapies. Moreover, the results obtained to date may also highlight important differences in immune circuits between COVID-19 and rheumatoid conditions that warrant further research in order to implement efficacious therapeutic regimens.

Therefore, the aim of this article was to review the existing evidence about immunomodulatory drugs that have been used in patients with COVID-19, including corticosteroids, antimalarials, anti-TNF, anti-IL-1, anti-IL-6, baricitinib, intravenous immunoglobulins, and colchicine, in order to inform about efficacious therapeutic indications and potential clues to COVID-19 pathogenesis. After an extensive research strategy, evidence was summarized as a narrative (non-systematic) review in order to provide an overall, updated state of the art as well as to inform non-structure expert opinion based on literature findings.

## 2. Pathogenesis

**Figure 1:**
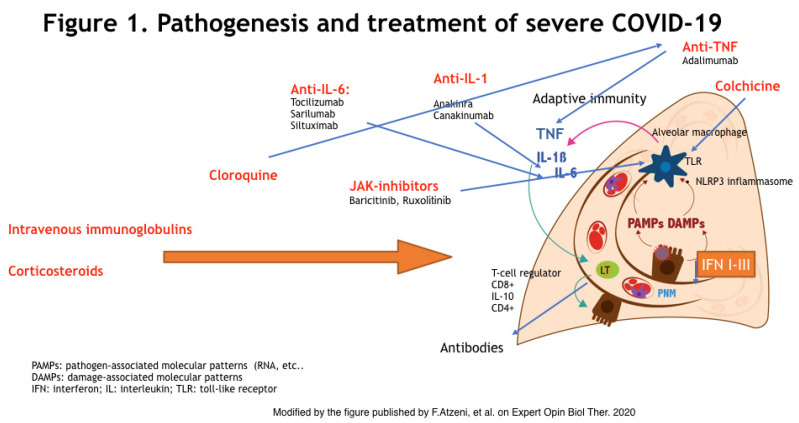
SARS-CoV-2-induced cytokine storm, the inflammatory pathway, and mechanisms of actions of the different drugs used to blocks this.

SARS-CoV-2 virus, as well as SARS-CoV-1 (coronavirus strain causing an outbreak in 2003), penetrates into the cells through the bond of the spike glycoprotein of the viral envelope with the angiotensin-converting enzyme 2 (ACE2), a receptor on the host cells’ surface [13]. ACE2 is a type I membrane protein expressed on type II pneumocytes, but it can be also found on renal, heart, gastrointestinal, and blood vessel cells [14]. SARS-CoV-2 infection leads to a wide range of pathogenic phenotypes, from asymptomatic individuals to COVID-19, which may exhibit severe clinical manifestations such as ARDS or MAS-like features. MAS causes hyperferritinemia, hepatic dysfunction, and disseminated intravascular coagulation (DIC) [15]. However, not all features of MAS are consistently found in COVID-19, as previously discussed, and COVID-19 seems to be associated with a relatively unique hyperinflammatory profile. Severe COVID-19 manifestations seem to be caused by a dysfunction of the immune system and to an uncontrolled release of several proinflammatory cytokines and chemokines. Furthermore, in patients with more severe clinical manifestations, higher levels of TNF-α, IL-1, IL-10, IL-6, and GM-CSF, among others, have been detected [10] (Figure 1).

The mechanisms underlying this uncontrolled release of inflammatory cytokines are not entirely clear to date. However, the replication rate of the virus can cause pyroptosis, that is, the inflammatory death of epithelial and endothelial cells, hence triggering the release of proinflammatory cytokines and chemokines [16]. This phenomenon also involves macrophages and lymphocytes and it may be the cause of peripheral lymphopenia in patients with severe COVID-19 [17]. Additionally, profound aberrations in the innate immunity have been described. Type I interferons (IFN-I) control viral replication and engage mediators of the adaptive response. In both SARS-CoV and MERS-CoV, the IFN-I response to the viral infection is either suppressed or profoundly impaired, hence causing an insufficient viral clearance together with a perpetuation of immune aberrations [18]. Moreover, the role of the adaptive immunity in this scenario cannot be entirely explained without considering the involvement of CD4^+^ and CD8^+^ T lymphocytes, since CD8^+^ cytotoxic T-cells (CTL) are able to secrete molecules such as perforine, granzymes, and INF-γ to eradicate the virus from the host cells [19], whereas helper CD4^+^ T lymphocytes (Th) assist CTL and B-cells by producing inflammatory cytokines and causing T-dependent B-cell activation [20].

IL-6 production can be directly induced by SARS-CoV-2, as well as by the stimulation of other cells of the immune system [21]. In fact, it has been proven that in COVID-19 infection, CD4^+^ T-cells are rapidly activated to differentiate into pathogenic Th1 cells, thus generating IFN-g and GM-CSF, among other proinflammatory cytokines. These mediators stimulate, in turn, the activation of monocytes, thereby causing high IL-6 release [22]. The activation of IL-6 is complex and requires the involvement of IL-6, its receptor (IL-6R) that is anchored to the membrane, and the gp130 co-receptor [23]. Nevertheless, many non-immune cells, including stromal and epithelial cells, can induce strong inflammatory responses when IL-6 and its soluble receptor attach to the membrane along with gp130 (trans-signaling), hence amplifying the inflammatory response [24].

IL-1 also plays an important role in both MAS and in COVID-19. IL-1 is a pleiotropic cytokine involved in inflammatory processes, hematopoiesis, and fibrosis [25]. The release of IL-1β depends on the activity of NLRP3 inflammasome, which responds to different noxious stimuli, including viral RNA [26]. Inflammasomes are large, multi-molecular complexes that are present on innate immunity cells, best known for their ability to control the activation of caspase-1, which, in turn, regulates the maturation of IL-1β and IL-18 [26]. It is increasingly evident that NLRP3 recognizes RNA viruses detecting cellular damage or distress induced by viroporins, pore-forming transmembrane proteins encoded by certain RNA viruses [27]. A recent study showed that the E protein of SARS-CoV creates channels that are permeable to Ca^2+^ ions and activates NLRP3 inflammasome [28]. It is likely that the mode of action of SARS-CoV-2 could be similar, hence supporting the elevated IL-1 concentrations observed in severe COVID-19 patients [29,30,31,32]. Moreover, IL-1β and TNF-α promote the response of Th17, by producing IL-17 [33], although their role in COVID-19 immunopathogenesis is still to be clarified.

In conclusion, profound alterations of the immune response in COVID-19 lead to an uncontrolled hyperinflammatory state that represents the main therapeutic target in this stage. Identifying an effective treatment strategy for critically ill patients with severe manifestations by immunomodulatory drugs is encouraged.

## 3. Clinical Presentation

COVID-19 presents with a wide spectrum of clinical phenotypes, including:

- Asymptomatic: percentages have not been clarified yet;
- Mild: (approx. 81% of the cases) or mild pneumonia [34];
- Severe: (approx. 14%) dyspnea, respiratory rate > 30 breaths/min, oxygen saturation (SpO2) < 93%, lung infiltrates > 50% in 24–48 h [35]; and
- Critical: (approx. 5%) multiple organ dysfunction [34], respiratory failure, septic shock.

The incubation period is characterized by a stable full blood count or mild leukopenia, increased viral load, and diffusion of the virus to ACE2-expressing tissues [4,36]. According to the WHO, current estimates indicate that SARS-CoV-2 could take between one to 14 days to incubate. Actually, a study from Taiwan showed that the SARS-CoV-2 median incubation period is about five days [6]. These estimates imply that, under conservative assumptions, 101 out of every 10,000 cases will develop symptoms after 14 days of active monitoring or quarantine [6]. The main symptoms in this phase include fever, which may not be very unresponsive to antipyretic drugs in some patients [35], cough, nasal congestion, general discomfort [35], headache, fatigue, myalgia, and conjunctivitis, apparently indistinguishable from any other respiratory infection. More severe signs and symptoms such as dyspnea are absent [35], and the involvement of the gastrointestinal system with diarrhea is rare.

After 7–14 days from the onset of the symptoms, an exacerbation may occur with a clinical picture of pneumonia: marked dyspnea [36], tachypnea (>30 breaths/min), hypoxemia (SpO2 < 90%), and severe lymphopenia with increased acute-phase proteins [5]. This phase is also associated with a state of hypercoagulability with initially normal levels of coagulation factors and D-dimer [36]. There is also an important increase of proinflammatory cytokines, including IL-2, IL-7, IL-10, G(M)-CSF, IP-10, MCP-1, MIP-1a, TNF-α, IL-1β, and IL-4 [25]. Patients with severe COVID-19 can show typical imaging features at the early stages of the disease. Chest CT plays a major role in the screening and early diagnosis of COVID-19 pneumonia [37,38]. A recent imaging study found that all patients had abnormalities in chest CT and most (98%) of them had bilateral involvement. The CT of COVID-19 patients has special characteristics, including (1) single or multiple ground-glass opacity (GGO), which is mainly a subpleural distribution; (2) crazy paving; (3) patchy GGO with segmental pulmonary consolidation; and (4) pulmonary consolidation [38]. Hospitalized patients with severe disease on admission were more likely to have bilateral multiple lobular and subsegmental areas of consolidation, while admitted patients with mild cases were more likely to have bilateral GGO and subsegmental areas of consolidation [38].

Finally, it is known that COVID-19 is not always confined to the respiratory tract and it may also invade the central nervous system through the hematogenous or retrograde neuronal route, inducing neurological symptoms or diseases such as headache, dizziness, impaired consciousness, ataxia, acute cerebrovascular disease, and epilepsy. Peripheral nervous system symptoms (hypogeusia, hyposmia, dysosmia, hypoxia, neuralgia), skeletal–muscular symptoms, dermatologic manifestations, and thrombotic manifestations were also described [39].

## 4. Research Strategy

The terms ‘SARS-CoV2’ or ‘COVID-19’, ‘lung involvement’, ‘storm cytokine’, ‘macrophage activation syndrome, ‘disseminated intravascular coagulation’, ‘treatment’, ‘therapy’, ‘corticosteroids’, ‘chloroquine’, ‘hydroxychloroquine’, ‘tocilizumab’, ’anakinra’, ‘anti-TNF drugs’, ‘canakinumab’, ‘intravenous immunoglobulins’, ‘baricitinib’, ‘colchicine’, and ‘management’ were used to search the Medline, PubMed, and Cochrane Library databases for English-language papers published between January 2020 and October 2020, and also to review the papers’ references and textbook chapters.

## 5. Therapy

The uncontrolled pulmonary and systemic inflammation is probably the leading cause of mortality in SARS-CoV-2 infection. While waiting for the development of specific antiviral therapies, monoclonal neutralizing antibodies and vaccines, which will be able to neutralize the etiological agent, an effective therapeutic strategy could be to counteract the hyperinflammatory state, which is responsible for the most severe clinical cases.

The cytokine profile of patients with severe COVID-19 is characterized by an increase of several cytokines, mainly (but not restricted to) IL-1β, IL-2, IL-6, IL-7, IL-8, and TNF-α [33].

Most of these mediators can be targeted with distinct drugs used in the rheumatology framework (Table 1), with attractive efficacy and safety profiles and, more importantly, with a long-lasting experience in the management of complex inflammatory scenarios.

### 5.1. Corticosteroids

Corticosteroids have been used for decades as therapeutic weapon for the treatment of numerous autoimmune diseases and to date they are still a fundamental aid. For instance, for the treatment of rheumatoid arthritis, the latest European League Against Rheumatism (EULAR) recommendations suggest the use of corticosteroids in flare-ups of disease and as therapeutic support during the initial phases of specific treatment, with the aim to more rapidly achieve remission and better inflammation control [3].

Corticosteroids are strong immunosuppressive drugs that act by blocking the NF-κB pathway, with consequent decreased transcription of a large number of proinflammatory genes.

In clinical practice, they are often used to treat viral pneumonia as a support to more specific therapies [40]. On the one side, these drugs may reduce the cytokine enhanced release, which is responsible for severe ARDS and multiple organ dysfunction. Although initial caveats around their potential effect on viral clearance, viral replication, and the risk of bacterial superinfections were raised, current evidence is solid about the beneficial effect of corticosteroids.

The majority of the information on their potential effectiveness has been extrapolated by data coming from their usage in flu, MERS, and SARS [41,42], and six RCT have already been carried out on COVID-19 patients [43,44,45,46,47].

A very recent meta-analysis including 15 studies carried out on patients with SARS, MERS, or COVID-19 (*n* = 5270) reported that the corticosteroid treatment is associated with higher mortality (RR = 2.11, 95% CI = 1.13–3.94, *p* = 0.019), longer hospital stays (weighted mean difference (WMD) = 6.31, 95% CI = 5.26–7.37, *p* < 0.001), higher rate of bacterial infections (RR = 2.08, 95% CI = 1.54–2.81, *p* < 0.001), and onset of hypokalemia (RR = 2.21, 95% CI = 1.07–4.55, *p* = 0.032) [39]. It needs to be highlighted that a confounding by indication bias cannot be excluded. Moreover, this finding has not been confirmed by a subset analysis where no correlation was observed between mortality and usage of corticosteroids in SARS-CoV (RR = 2.56, 95% CI = 0.99–6.63, *p* = 0.053) and in MERS-CoV (RR = 2.06, 95% CI = 0.66–6.44, *p* = 0.213, *p* = 0.002). In addition, the use of corticosteroids was not homogeneous within the studies that were considered in the meta-analysis, in terms of dosage, start of administration, and treatment duration [42].

Russel et al. [41] do not support the use of corticosteroids in patients with SARS-CoV-2-induced pneumonia. By contrast, Zhou et al. [48] showed a favorable attitude to the use of corticosteroids. They analyzed clinical data of 15 patients with SARS-CoV-2 infection, who were admitted to the intensive care unit (ICU) of Wuhan Pulmonary Hospital. In patients with bilateral pneumonia, hypoxemia, and moderate/severe ARDS with no response to the administered therapies, a treatment with corticosteroids (median hydrocortisone equivalent dose of 400.0 mg/day) was started as soon as the patients were admitted in the ICU, and it was continued for an average of 9.5 days. A benefit was not seen in terms of mortality, but during the first 3–5 days of systemic corticosteroid therapy, an improvement of the partial oxygen pressure (PaO_2_) and of the PaO_2_/FiO_2_ ratio (fraction of inspired O_2_) was registered. Invasive mechanical ventilation also certainly contributed to obtaining this improvement. The authors hypothesized a positive effect of corticosteroids, together with other treatments, in patients with severe ARDS, mainly in moderating the hyperinflammatory state and consequently reducing the risk of multiple organ damage and shock [49]. However, they underlined that corticosteroids should only be taken into account for patients with moderate/severe ARDS, sepsis, and shock. The authors suggest a dose of methylprednisolone < 1 mg/kg of body weight for a duration not greater than seven days and only in patients with moderate/severe ARDS [49].

Moreover, higher doses seem to promote the onset of adverse effects, such as psychosis, hyperglycemia, avascular necrosis, and secondary infections [50].

In addition, their early usage in patients with mild symptoms, aside from being not recommended for the above reasons, may also be ineffective. An observational study carried out by Lei Zha et al. [51] evaluated the viral clearance and the clinical course in 31 patients hospitalized for mild SARS-CoV-2 infection. These patients presented with more clinical symptoms, for example, cough, myalgia, fever, higher inflammatory indices, and greater CT abnormalities, hence suggesting that the benefit of corticosteroid usage could be correlated with the severity of the symptoms at presentation.

Interestingly, it was observed that the treatment with corticosteroids did not influence the viral clearance (different from what was suspected) and it neither modified the hospitalization time nor the duration of symptoms in patients with mild COVID-19 [44].

To date, the best approach is probably to use no corticosteroids systematically in all patients with COVID-19, as also recommended by the WHO, but to reserve their use particularly for more severely affected patients. In fact, there is evidence that dexamethasone usage reduced mortality in several groups of patients [43,45] (including those requiring oxygen therapy or the elder subgroups). In this context, a trial conducted on patients with moderate/severe ARDS, with the aim to evaluate the effectiveness of corticosteroids to reduce mortality and duration of invasive mechanical ventilation, has produced encouraging results so far [48]. The recent Randomised Evaluation of COVID-19 therapy (RECOVERY) trial, a randomized, controlled, open-label, adaptive, platform trial on 2104 patients randomly allocated to receive dexamethasone compared with 4321 patients concurrently allocated to standard of care. This study showed that 454 (21.6%) patients allocated dexamethasone and 1065 (24.6%) patients allocated to usual care died within 28 days (age-adjusted rate ratio [RR] = 0.83; 95% CI = 0.74–0.92; *p* < 0.001) [43]. The proportional and absolute mortality rate reductions varied significantly depending on level of respiratory support at randomization. In particular, dexamethasone reduced deaths by one-third in patients receiving invasive mechanical ventilation (29.0% vs. 40.7%, RR = 0.65 [95% CI = 0.51–0.82]; *p* < 0.001) and by one-fifth in patients receiving oxygen without invasive mechanical ventilation (21.5% vs. 25.0%, RR = 0.80 [95% CI = 0.70–0.92]; *p* = 0.002) but did not reduce mortality in patients not receiving respiratory support at randomization (17.0% vs. 13.2%, RR = 1.22 [95% CI = 0.93–1.61]; *p* = 0.140). Finally, in patients hospitalized with COVID-19, dexamethasone reduced 28-day mortality among those receiving invasive mechanical ventilation or oxygen at randomization, but not among patients not receiving respiratory support [43]. Overall, subsequent randomized clinical trials supported these findings [52], thus concluding that corticosteroids are an effective, inexpensive, and safe (first-line) treatment in severe COVID-19.

### 5.2. Chloroquine and Hydroxychloroquine

Chloroquine is an old drug used in the treatment and prophylaxis of malaria and other intracellular bacteria such as *Coxiella burnetii* and *Tropheryma whipplei* [53]. Hydroxychloroquine differs from chloroquine only for the presence of a hydroxyl group at the extremity of the side chain and, besides acting against intracellular agents, it has also an important immunomodulatory role, hence its common use in rheumatology to treat several conditions.

Chloroquine has been taken into account because earlier studies on other coronaviruses, including SARS-CoV-1, showed an important antiviral action in vitro [54,55].

Chloroquine modes of action are numerous. It can reduce the ACE2 glycosylation, as proven in studies carried out on SARS-CoV, thus compromising the viral entry. It can also affect another early phase of the viral replication, interfering with the pH-dependent viral entry mediated by the endosome, as it was studied for the Dengue or Chikungunya viruses [56,57]. Another potential mechanism consists of inhibiting the viral release into the intracellular space.

Inside the host cell, SARS-CoV virions are surrounded by a vesicle derived by the cellular membrane (endosome), where they replicate. The activation phase occurring in the endosomes with acid pH provokes the fusion of the viral and endosomal membranes, leading to the SARS-CoV1 genome release to the cytosol [58]. In absence of antiviral drug, the virus is directed to the lysosomal compartment, where the low pH, together with the action of enzymes, breaks the viral particle, releasing the viral nucleic acid and often also the enzymes necessary for its replication [59]. Chloroquine is thought to accumulate in the lysosomes, interrupting the normal lysosome–endosome fusion process, thereby inhibiting the release of the viral content [56].

Furthermore, an additional mode of action concerns chloroquine immunomodulatory ability. In fact, it would seem that both chloroquine and hydroxychloroquine are able to reduce IL-1 and IL-6 levels [60]. Moreover, it has also been noticed that chloroquine is able to inhibit the production of TNF by immune cells [61]. These findings are particularly important in the context of COVID-19 infection, in order to abrogate the enhanced cytokine production. These mechanisms provide strong support to the hypothesis that hydroxychloroquine could also be able to promote the ability to suppress the hyperactivation of the immune system triggered by SARS-CoV-2 infection and slow down the progression from mild to severe disease [62].

Hydroxychloroquine has an action that is similar to that of chloroquine, but it is better tolerated and more available worldwide. For this reason, the Chinese as well as other countries’ guidelines consider these two drugs in different dosages: chloroquine at a dose of 500 mg two times a day (BID) for 10 days or, alternatively, hydroxychloroquine at a dose of 200 mg (BID) [55].

Multicentric clinical studies carried out in China have shown that the treatment with chloroquine causes a clinical improvement of several patients’ outcomes and reduces the hospital stay without increasing the adverse effects [59]. However, further RCTs have not confirmed these beneficial effects at any stage of the disease [63,64,65,66].

Then, a randomized, double-blind, placebo-controlled trial was conducted in the United States and in Canada to evaluate the use of hydroxychloroquine as post-exposure prophylaxis. The authors enrolled a total of 821 patients who had been in close contact with a person with a confirmed COVID-19 diagnosis in the previous four days. Participants were randomized to receive HCQ or placebo and then monitored with online surveys. The authors then analyzed the incidence of laboratory-confirmed or symptoms consistent with SARS-CoV-2 infection in the following 14 days. SARS-CoV-2 infection developed in 107 of 821 participants (13.0%) during the follow-up, with a similar incidence in the two groups. The authors concluded that HCQ is not effective as post-exposure prophylaxis, while acknowledging several limitations in their study, including the impossibility for most patients to access the PCR or serologic testing in absence of symptoms, limiting the ability to identify asymptomatic infections [67].

Of note, studies on hydroxychloroquine suffer from poor methodological robustness. In fact, heterogeneous standards emerge, which are not rigorously considered when reporting the data. Moreover, it must be noted that these drugs are contraindicated in G6PD-deficient patients, as well as in patients with impaired heart function due to their effect on QT prolongation.

### 5.3. Anti-Tumor Necrosis Factors

In COVID-19 patients, high concentrations of TNF-α seem to correlate with the severity of the clinical picture [62]. Relying on the information obtained from SARS-CoV, the increase of TNF-α seems to be due also to the up-regulation of TACE induced by the virus itself, which results in the shedding of the terminal carboxylic intracytoplasmic component of ACE, leading to the formation of soluble ACE. The function of soluble ACE is largely unknown, but it is believed that it could contribute directly to the inflammatory process. In fact, the increase of soluble ACE is associated with increased TNF-α production [68].

Based on these lines of evidence, the use of anti-TNF-α monoclonal antibodies, largely employed for autoimmune diseases, has been proposed in COVID-19 with the aim of inhibiting this cytokine and thus reducing the inflammatory effects triggered by this cytokine.

Despite this pathogenic rationale, evidence of TNF blockade in COVID-19 is limited. One study registered on the “Chinese Clinical Trial Registry” (ChiCTR2000030089) designed to evaluate the effectiveness of adalimumab (at unspecified dose) in COVID-19 infection has been indexed [69], and a non-controlled, observational study reported no clinical benefit [70].

### 5.4. Anti-Interleukin-1

Interleukin-1 (IL-1) is another cytokine that seems to play a crucial role in the hyperinflammation, cytokine storm, and even secondary hemophagocytic lymphohistiocytosis (sHLH).

In this respect, from the re-analysis of a phase 3 randomized controlled trial evaluating the effectiveness of anakinra (a recombinant human IL-1 receptor antagonist) in patients with sepsis, it was observed that it leads to benefits in terms of survival in patients with MAS [71]. However, although initially neglected, it must be noted that divergences in the immunopathogenesis of MAS/sHLH and COVID-19 have been highlighted.

Three trials have been approved to explore the efficacy of anakinra in these conditions. The first of them is designed as a three-arm study, with the aim to evaluate the effectiveness and safety of anakinra or emapalumab (an anti-IFNγ monoclonal antibody used for MAS [72]) against the standard therapies, in patients with respiratory failure and clinical signs of MAS (NCT04324021)

The ESCAPE Clinical Trial, recently approved, is a study with the purpose of evaluating the efficacy of a personalized immunotherapy. The study design includes two arms, one of them using anakinra (200 mg three times daily for seven days in patients with characteristics of MAS), the other one using tocilizumab (8 mg/kg body weight once up to a maximum of 800 mg in patients with characteristics more of an immune system dysregulation) (NCT04339712) [73]. Finally, the NCT04330638 trial has a more complex design, whose main goal is to compare the efficacy of IL-1 blockade, IL-6 blockade, and combined IL-1 and IL-6 blockade. Consequently, this study includes the following arms: patients treated only with anakinra (daily subcutaneous injection of 100 mg for 28 days), only with tocilizumab (single IV infusion at dose of 8 mg/kg with a maximum infusion of 800 mg/injection) or siltuximab (single IV infusion at dose of 11 mg/kg), with the combination of anakinra and tocilizumab (single IV infusion at dose of 8 mg/kg with a maximum infusion of 800 mg/injection plus daily subcutaneous injection of 100 mg for 28 days), and with the combination of siltuximab and anakinra (single IV infusion of siltuximab at a dose of 11 mg/kg + daily subcutaneous injection of 100 mg for 28 days) [74]. Tocilizumab and siltuximab are both anti-IL-6 monoclonal antibodies [75].

However, although real-life, published studies report some beneficial effect in some outcomes, overall, they do not support a consistent effect on mortality, ventilation, or WHO clinical improvement [76,77,78]. Furthermore, a multicenter, open-label, Bayesian randomized clinical trial (CORIMUNO-ANA-1), nested within the CORIMUNO-19 cohort, in which patients from 16 university hospitals in France with mild-to-moderate COVID-19 pneumonia, severe acute respiratory syndrome coronavirus 2 infection confirmed by real-time RT-PCR, were treated with anakinra, showed that in the analyzable population, the median age was 66 years (IQR 59 to 76) and 80 (70%) participants were men. In the anakinra group, 21 (36%) of 59 patients had a WHO-CPS score of more than 5 at day four versus 21 (38%) of 55 in the usual care group (median posterior absolute risk difference [ARD]—2.5%, 90% credible interval [CrI]—17.1 to 12.0), with a posterior probability of ARD of less than 0 (i.e., anakinra better than usual care) of 61.2%. At day 14, 28 (47%; 95% CI 33 to 59) patients in the anakinra group and 28 (51%; 95% CI 36 to 62) in the usual care group needed ventilation or died, with a posterior probability of any efficacy of anakinra (hazard ratio [HR] being less than 1) of 54.5% (median posterior HR 0.97; 90% CrI 0.62 to 1.52). At day 90, 16 (27%) patients in the anakinra group and 15 (27%) in the usual care group had died. Serious adverse events occurred in 27 (46%) patients in the anakinra group and 21 (38%) in the usual care group (*p* = 0.45). To conclude, anakinra did not improve outcomes in patients with mild-to-moderate COVID-19 pneumonia [79]. Finally, the Italian Medicines Agency (AIFA) has approved the compassionate use of canakinumab to neutralize the cytokine release in COVID-19 patients with severe pulmonary involvement [80]. Canakinumab is an anti-IL-1 monoclonal antibody totally humanized (the proposed canakinumab dose is 600 mg in 250 mL of 5% dextrose infused intravenously (i.v.) over 2 h), approved for the treatment of the familial Mediterranean fever [81].

### 5.5. Anti-Interleukin-6

Another cytokine that appears to play a key role in this hyperinflammatory condition is IL-6 [82]. As previously reported, its increased plasma concentration in COVID-19 patients is accompanied by an increase of CRP and development of lymphopenia [83]. Taken together, these conditions indicate a turn toward a severe manifestation of the SARS-CoV-2 infection. Similar to IL-1, IL-6 seems to play a key role both in the development of ARDS and in the development of MAS-like manifestations.

However, some authors believe that IL-6, especially in the initial stages of infection, plays an important role in suppressing the viral replication. Consequently, an early pharmacological blockade of this cytokine may negatively contribute to the viral clearance [83]. The real correct timing for the administration of drugs against IL-6 needs to be established from the numerous clinical trials that have been launched.

Probably the best time would be when the clinical and laboratory signs related to the hyperinflammation stage begin to appear. In particular, these signs would be the reduction of CD4^+^ and CD8^+^ T-cells, the increase of inflammation indices, hyperferritinemia, the increase of IL-6, and the appearance of thrombocytopenia [84,85].

Promising results on the use of tocilizumab, humanized IgG1 monoclonal antibody directed against the human IL-6 receptor, in patients with severe manifestations of COVID-19 have already been observed in data collected from a small retrospective study involving 21 tocilizumab-treated patients with severe COVID-19. In this study, an improvement of lung lesions on chest CT, of plasma oxygenation and lymphopenia, and a reduction of the inflammation indices were found [86].

To date, numerous trials have been approved and/or started to evaluate the effectiveness of anti-IL-6 in patients with severe manifestations of COVID-19. Tocilizumab, sarilumab (a human monoclonal IgG1 antibody, which binds specifically to both soluble- and membrane-bound IL-6 receptors), and siltuximab (an anti-IL-6 chimeric IgG1k monoclonal antibody) are/will be evaluated in several clinical trials, both alone and in combination with other treatments (clinicaltrials.gov).

In particular, one of the first multicentric, randomized, clinical trials on tocilizumab in patients with COVID-19 pneumonia and high IL-6 levels was approved in China (ChiCTR2000029765) [87]. There are currently 41 clinical trials with tocilizumab for COVID-19 registered in clinicaltrials.gov.

In Italy, AIFA has approved a phase 2 multicentric study to evaluate the efficacy of tocilizumab in patients with severe COVID-19 pneumonia (TOCIVID-19) [88,89]. Also, the Spanish Medicines Agency (AEMPS) has approved another phase 2, called COVITOZ-01 [90].

Nevertheless, Chinese and Italian guidelines have introduced the use of tocilizumab for the treatment of COVID-19. The tocilizumab dose suggested in the various clinical trials is 8 mg/kg i.v. However, data on safety and efficacy of tocilizumab in COVID-19 are currently controversial and inconclusive. Three studies from Italy report on retrospective studies on tocilizumab in COVID-19 using historical controls [91,92,93]. However, the inclusion criteria for treating with tocilizumab were unclear, which may explain this controversy. Recently, Roche announced the closure of the first global, randomized, double-blind, placebo-controlled phase III COVACTA trial of tocilizumab in hospitalized patients with severe COVID-19-associated pneumonia because of failure to meet the primary endpoint of improved clinical status. However, a recent randomized clinical trial has reported that tocilizumab reduced the likelihood of progression to a composite outcome of mechanical ventilation or death, but it did not improve survival in COVID-19 patients [94]. Furthermore, REMAP-CAP trial shows decreased mortality in ICU patients (35% vs. 28%) [95]. IL-6 levels are associated with ARDS in critically ill patients. Although levels of IL-6 are not as elevated as sepsis or ARDS, this cytokine plays a key role in the pathogenesis of COVID cytokine storm [96]. Taken together, these findings may suggest that tocilizumab may provide some beneficial effect in some COVID-19 patients, but whether it is clinically relevant or how to identify patients (and concomitant therapies) who will benefit the most are to be elucidated.

### 5.6. Baricitinib

Among the drugs for rheumatic conditions, baricitinib (a JAK1 and JAK2 inhibitor) has aroused interest in the treatment of COVID-19 patients, as it seems to be able to reduce the endocytosis of the virus at cellular level. As previously reported, once SARS-CoV-2 binds to ACE2, it undergoes cellular internalization by endocytosis. Baricitinib seems to be able to inhibit both AAK1 (AP2-associated protein Kinase 1) protein and Cyclin G-associated kinase (GAK), molecules both involved in the mechanism of viral endocytosis [97,98] as well as in the intracellular assembly of the virus particles [98]. Therefore, baricitinib may not only limit viral endocytosis but also it may help in managing the uncontrolled cytokine release observed in COVID-19. In fact, due to its effects on the inhibition of the JAK-STAT pathway [99], baricitinib treatment leads to a strong reduction in the production of a number of cytokines, including IL-6 and IFNγ [95,96]. Taken together, based on the multi-level, complimentary effects of baricitinib for viral infection process as well as its strong inflammatory response, the use of baricitinib may be supported in some stages of COVID-19.

Despite these promising findings on the potential efficacy of baricitinib, other aspects lead to a limitation in its use. First, it is important to remember that type I IFN production is one of the initial and pivotal responses of the innate immunity against viral replication, especially in the earliest stages of infection [100]. Since type I IFN signaling depends on the JAK/STAT pathway via JAK1, baricitinib treatment might somehow reduce the type I IFN-mediated antiviral response [101,102].

Moreover, baricitinib treatment cannot be initiated in patients with a lymphocyte count lower than 0.5 × 10^9^ cells/L [103]. Importantly, ICU patients usually exhibit these counts, and lymphocytopenia may be a risk factor for disease progression in COVID-19. Additionally, patients who did not survive COVID-19 are likely to suffer from anemia, and baricitinib may further aggravate this condition [104].

Based on these data, it can be inferred that the use of baricitinib in patients with COVID-19 is controversial. Robust studies are needed to evaluate the efficacy, safety profiles, and optimal timeframes for this drug in COVID-19.

Currently, there are different clinical trials approved for the study of baricitinib in patients with COVID-19. The NCT04340232 clinical trial is a phase 2/3 study that will be conducted in Colorado with the aim of evaluating the safety of baricitinib in phase 2, with the enrollment of 20 patients, and in phase 3, the subsequent evaluation of the efficacy of baricitinib in patients with COVID-19, with the enrollment of an additional 60 patients [105].

The clinical trial NCT04320277 launched in Italy at Prato hospital will evaluate the efficacy of baricitinib in combination with anti-viral therapy in patients with mild and moderate COVID-19 [106].

Finally, the recently approved clinical trial NCT04321993 in Scotland is a phase 2 study divided into several arms. Each arm will evaluate a different treatment that will be offered to all hospitalized patients with moderate and severe COVID-19. Among the various treatments proposed, there is baricitinib. Certainly, the first results of these clinical trials will be able to clarify what is the role of baricitinib in the treatment of COVID-19 [107].

A recent randomized clinical trial enrolling more than 1000 patients reported that baricitinib (in combination with remdesivir) has beneficial effects on reducing recovery time and accelerating improvement in clinical status among patients with COVID-19, notably among those receiving high-flow oxygen or noninvasive ventilation. However, no differences were noted in mortality [108]. Further validation and the evaluation of the clinical significance of these findings are warranted.

### 5.7. Intravenous Immunoglobulins

Although antibodies play a crucial role in mediating protection against viral infection, their role on the clearance of established SARS-CoV2 infection and clinical outcomes is less evident [109]. According to the pathogenesis, it has also been hypothesized that neutralizing antibodies (NAbs) could have a possible harmful role. In fact, the antibody-dependent enhancement of viral infections has been largely studied in virology [110]. Thanks to information extrapolated from the pathogenetic mechanism of SARS-CoV infection, it has been seen that in 80% of patients who developed lung complications, there was a concomitant increase in the concentration of NAb IgG [111]. Probably these patients develop an early but suboptimal antibody response, with the formation of antibodies that are not able to neutralize the virus effectively. However, these antibodies bind to the virus, and the virus-NAb complexes bind to the Fc receptors (FcR) on the surface of macrophages.

On the one hand, this bond leads to the endocytosis of the virus with consequent viral replication inside the cells, thus potentially contributing to the severity of the disease. On the other hand, it allows the activation of the intracellular enzymatic cascade, thus leading to the activation of the transcription of genes encoding proinflammatory cytokines, thus enhancing cytokine release [83]. However, limited evidence is available regarding the expression of cytokines and chemokines in SARS-CoV infection [112]. Although confirmed in animal models, the exact contribution to human disease is uncertain, with results ranging from protective to disease-enhancing [110]. However, it must be noted that the detrimental effects of antibodies are not strictly related to the level of neutralizing antibodies but also on their neutralization potency, which is also associated with cytokine signatures [109] This adds another layer of complexity to this scenario and urges the simultaneous evaluation of antibody responses (either by production or passive transfer) and cytokine serum milieu in order to better understand and predict clinical outcomes.

Furthermore, the virus-NAb complexes may also activate the classical complement pathway. According to this data, it has been hypothesized that preventing FcR from binding to the virus-Nab complexes may abrogate these negative effects. The intravenous immunoglobulins (IVIg) may competitively inhibit this bond, occupying the FcR, which in turn will be less likely to be available for binding to the virus-NAb complexes [103].

Lin et al. [83] support the use of IVIg in the treatment of COVID-19 patients with severe clinical presentation when the onset of lymphopenia is observed. In fact, this condition frequently occurs in patients who progress toward a severe pulmonary involvement [108]. Specifically, these authors indicate the best time for administration is the moment when the following conditions are observed: reduction of B- and T-cells, increase of inflammatory cytokines such as IL-6, increase of CRP, increase of D-dimer in peripheral blood, and the worsening of the lung imaging on CT. Moreover, they suggest the concomitant use of low-molecular-weight heparin as anticoagulant therapy to counteract the onset of DIC [81]. Regarding dosage, this group recommends the use of high-dose IVIg (0.3–0.5 g/kg of body weight for five days), as this could interrupt the cytokine-enhanced release at an early stage [83].

Three clinical trials have been currently approved to evaluate the efficacy of IVIg (0.5 g/kg/d for five days) in patients with severe SARS-CoV-2 infection (NCT04261426) [113].

Finally, exogenous neutralizing antibodies were demonstrated to be efficacious in controlling viral infection several years ago, although their title rapidly declined, allowing reinfection [114]. The downside of antibodies is that it appears that both in seasonal coronaviruses as well as in SARS-CoV and MERS, there is a rapid decline of antibody titers and neutralizing activity [115]. Infusion of human neutralizing antibodies into animals provided protection against SARS and SARS-CoV-2 [116]. Preliminary data showed that infusion of plasma from convalescent COVID-19 patients might be helpful in patients with severe disease, but evidence is scarce. Randomized trials are needed and a better appraisal of the serum cytokine signatures is imperative in order to better delineate the connections with clinical outcomes.

### 5.8. Colchicine

Another drug of recent interest for the treatment of patients with COVID-19 is colchicine. This drug is widely used in the treatment and prophylaxis of acute gout attacks [117] and in other articular pathologies caused by microcrystals [118]. It is also used in the treatment of Familial Mediterranean Fever [119], and in the EULAR guidelines it is indicated for the treatment of oral and genital ulcers in patients with Behçet disease [120].

Colchicine is a drug with a powerful anti-inflammatory action. The mechanisms through which it carries out its own anti-inflammatory action are manifold, including inhibition of the NLRP3 inflammasome, impairment of cytoskeleton functionality (preventing mitosis, cell motility, adhesion, chemotaxis, and viral internalization), and reducing inflammatory response, especially in granulocytes and monocytes.

Based on these reasons, the use of colchicine in patients with COVID-19 has been hypothesized, hoping that it will be able to block virus entry and counteract the hyperinflammatory state.

Several clinical trials have already been authorized in different countries for testing this drug in this condition (NCT04350320, NCT04326790, NCT04328480, NCT04322565, NCT04322682) (doses varied according to the trials, with a median dose of 0.5–1 mg/daily).

Recently, a randomized, double-blind trial involving non-hospitalized patients with COVID-19 diagnosed by polymerase chain reaction (PCR) testing or clinical criteria involving a total of 4488 patients treated with colchicine (0.5 mg twice daily for three days and once daily thereafter) or placebo showed that the composite of death or hospitalization for COVID-19 occurred in 4.7% of the patients in the colchicine group and 5.8% of those in the placebo group (odds ratio, 0.79; 95.1% confidence interval (CI), 0.61 to 1.03; *p* = 0.08) [121]. Among the 4159 patients with PCR-confirmed COVID-19, the composite of death or hospitalization for COVID-19 occurred in 4.6% and 6.0% of patients in the colchicine and placebo groups, respectively (odds ratio, 0.75; 95% CI, 0.57 to 0.99; *p* = 0.04). In the patients with PCR-confirmed COVID-19, the odds ratios were 0.75 (95% CI, 0.57 to 0.99) for hospitalization due to COVID-19, 0.50 (95% CI, 0.23 to 1.07) for mechanical ventilation, and 0.56 (95% CI, 0.19 to 1.66) for death. Serious adverse events were reported in 4.9% and 6.3% in the colchicine and placebo groups (*p* = 0.05). Pneumonia occurred in 2.9% and 4.1% of patients (*p* = 0.02). Diarrhea was reported in 13.7% and 7.3% in the colchicine and placebo groups (*p* < 0.0001) [121]. To conclude, among non-hospitalized patients with COVID-19, colchicine reduced the composite rate of death or hospitalization. However, it is necessary to wait for the results of further clinical trials to evaluate its effectiveness and understand what the effective dosages and the correct timing to start the treatment are.

## 6. Conclusions

No antiviral or anti-inflammatory treatments have been shown to be clearly effective for SARS-CoV-2 infection. In the presence of the patient’s respiratory deterioration or even earlier, if there are laboratory data on hyperinflammatory state, a short course of high-dose intravenous corticosteroids will provide an important clinical benefit. In certain patient groups, a single dose of tocilizumab may avoid patient transfer to the ICU or the need for invasive ventilation. There is insufficient, good-quality evidence for most of the drugs used in the rheumatology framework, although treatment with anakinra, anti-TNF, or antimalarials does not seem advisable. Whether other immunomodulatory therapies such as baricitinib, IVIg, or colchicine may be of some clinical utility remains to be clarified in current trials (Table 2).

Other therapies (e.g., antimalarials, anti-TNF agents, baricitinib, intravenous immunoglobulin, colchicine) are currently being studied with variable rates of success and may be clinically useful.

To date, taking into account published studies, no antiviral or anti-inflammatory treatment has been shown to be clearly effective for SARS-CoV-2 infection and, unfortunately, several studies have shown contradictory results. Due to the high number of infected people and the high lethality index in the context of the present SARS-CoV-2 world pandemic, the majority of the clinical and therapeutic decisions have been made based on the experience instead of the evidence. To all this must be added the need to reduce the number of patients who have required mechanical ventilation and admission to ICU (due to the limited number of beds in ICUs).

The frenzy to desperately respond to the COVID-19 pandemic made us forget the principle of Galilean research and has led now, almost a year after the appearance of the virus, to have inconclusive results on the efficacy of several therapeutic approaches. Too many observational, largely heterogeneous, and powerless studies have been conducted and almost no randomized, double-blind, robust clinical trials have seen the light. The lack of control groups and the different timing of administration of these treatments during infection are the two most important causes that may explain the contradictory results among the different studies. Heterogeneity among studies has added another layer of complexity, thus impeding efficient meta-analyses and validation. Taken together, it seems that high-dose corticosteroids and probably tocilizumab are treatments from the rheumatology framework that could work in COVID-19 patients in rescuing patients from respiratory deterioration, hyperinflammation, or transfer to ICU.

Specifically, the critical point in treating the inflammatory phase of COVID-19 is deciding when and how to use anti-inflammatory therapies. Currently, no clear clinical or biochemical markers are validated to determine which patients might benefit from immune-suppressive treatment. In particular, in regard to tocilizumab, the accurate selection of the patients may play a pivotal role in determining the successful therapy outcome. IL-6 levels could be a surrogate marker to identify the presence of a hyperinflammatory response, and D-dimers values might be also helpful. Although integrative algorithms with different biomarkers have been proposed, these findings suffer from the same flaws as therapeutic trials, and clinical validation should be established. Another therapeutic option to explore could be the association of tocilizumab with an antiviral drug. Randomized clinical trials are needed to elucidate all these issues.

In regard to chloroquine and hydroxychloroquine, although results were promising, current results do not seem to support these drugs in COVID-19. HCQ has apparently failed as post-exposure prophylaxis, but its efficacy on earlier time frames is unknown. It must be noted that treatments of influenza with oseltamivir as well as treatment of varicella zoster with acyclovir need to be started within a few hours to be clinically efficacious. Trials analyzing HCQ as preemptive therapy in high-risk groups could lead to more effective results, although the risk of adverse events needs to be considered with caution.

In addition to pharmacological treatments, in the short term, passive transfer of neutralizing antibodies represents both a prevention and a treatment option. Engineered neutralizing antibodies with enhanced antiviral activity and extended life span might be also a valuable tool in the hands of clinicians, in terms of prevention and treatment.

The clinical trial research has occurred at an unprecedented pace during these last few months. Historically, certain attributes of clinical trials, including time-consuming recruitment and consent processes, limited generalizability, and exorbitant costs, have limited their capacity to provide rapid answers to important clinical questions. Today, the COVID-19 pandemic has induced major changes to the infrastructure of clinical trials that have transformed and improved their potential to generate high-quality evidence with variable success. These major changes align with the domains of a ‘threshold concept’, such as transformative, troublesome, integrative, and discursive, among others, which should be tackled beyond clinical care.

In conclusion, an accurate early detection and treatment of possible complications with a multidisciplinary approach could allow a better recovery of patients from ARDS due to COVID-19.

## Figures and Tables

**Table 1:** Laboratory parameters and rheumatological indications of the drugs used to treat COVID-19.

Drugs	Rheumatological Indications	Laboratory Parameters	Contraindications
**Chloroquine and hydroxychloroquine**	RA, SLE, SS	Cytopenia, long QT	Maculopathy, retinal changes, hypersensitivity, G6PD deficiency
**IVIG**	Off-label	Decrease in Hb levels, positive Coombs test, increase in blood lactate dehydrogenase and liver enzyme levels	Hypersensitivity, type I or II hyperprolinemia
**Anti-TNF drugs**	RA, JIA, SpA, PsA, BD	Cytopenia, increase in ALT levels, prolongation of activated partial thromboplastin time, auto-antibody test positivity (including double stranded DNA antibodies),	Hypersensitivity, active tuberculosis or other severe infections, NYHA III IV
**Anti- IL 6 drugs**	RA, JIA, cytokine release syndrome, GCA	Increase in liver enzyme levels, decrease in neutrophil and platelet counts	Hypersensitivity, active severe infections
**Anti- IL 1 drugs**	RA, CAPS	Decrease in neutrophil and platelets counts, increase in eosinophil differential percentage	Hypersensitivity
**Baricitinib**	RA	Decrease in neutrophil and lymphocyte counts, decrease in Hb levels, alterations in lipid parameters, increase in liver enzyme and creatine phosphokinase levels	Hypersensitivity, pregnancy
**Colchicine**	Gout, Familial Mediterranean Fever, BD	Decrease in neutrophil and lymphocytes, hepatic/renal function alteration	Hypersensitivity

RA: rheumatoid arthritis; JIA: juvenile rheumatoid arthritis; SpA: spondyloarthritis; PsA: psoriatic arthritis; SLE: systemic lupus erythematosus; SS: Sjögren syndrome; CAPS: cryopyrin-associated periodic syndrome; G6PD: glucose-6-phosphate dehydrogenase; BD: Behçet disease; GCA: giant cell arteritis.

**Table 2:** Take-home messages.

Take-home messages
The altered immune response against SARS-CoV-2 that leads to an uncontrolled hyperinflammatory response is a major therapeutic target.
In patients hospitalized with COVID-19, dexamethasone reduced 28-day mortality among those receiving invasive mechanical ventilation or oxygen at randomiza-tion, but not among patients not receiving respiratory support.
High-dose, intravenous methylprednisolone and a single dose of tocilizumab may avoid patient transfer to an ICU and the need for invasive ventilation.
In placebo-controlled trials in hospitalized patients with severe COVID-19-associated pneumonia, tocilizumab failed to meet the primary endpoint of im-proved clinical status and the key secondary endpoint of reduced patient mortali-ty, but time frames may be crucial for treatment outcomes.
